# Hibernation Leads to Altered Gut Communities in Bumblebee Queens (*Bombus terrestris*)

**DOI:** 10.3390/insects9040188

**Published:** 2018-12-07

**Authors:** Lien Bosmans, María I. Pozo, Christel Verreth, Sam Crauwels, Felix Wäckers, Hans Jacquemyn, Bart Lievens

**Affiliations:** 1 Laboratory for Process Microbial Ecology and Bioinspirational Management (PME&BIM), Department of Microbial and Molecular Systems, KU Leuven, Campus De Nayer, B-2860 Sint-Katelijne-Waver, Belgium; lien.bosmans@kuleuven.be (L.B.); christel.verreth@kuleuven.be (C.V.); sam.crauwels@kuleuven.be (S.C.); 2 Plant Conservation and Population Biology, Biology Department, KU Leuven, B-3001 Heverlee, Belgium; maribel.pozoromero@kuleuven.be (M.I.P.); hans.jacquemyn@kuleuven.be (H.J.); 3 Biobest Group, B-2260 Westerlo, Belgium; felix.wackers@biobestgroup.com; 4 Lancaster Environment Centre, Lancaster University, Lancaster LA1 4YQ, UK

**Keywords:** *Bombus terrestris*, gut microbiota, hibernation, ileum, midgut, queen

## Abstract

Many reptiles, amphibians, mammals, and insects practice some form of hibernation during which their metabolic rate is drastically reduced. This allows them to conserve energy and survive the harsh winter conditions with little or no food. While it can be expected that a reduction in host metabolism has a substantial influence on the gut microbial community, little is known about the effects of hibernation on the composition of the microbial gut community, especially for insects. In this study, we assessed and compared the bacterial gut community composition within the midgut and ileum of indoor-reared queens of *Bombus terrestris* before and after an artificial hibernation period of 16 weeks. Deep sequencing of 16S ribosomal RNA gene amplicons and clustering of sequence reads into operational taxonomic units (OTUs) at a similarity threshold of 97% revealed several bacterial taxa that are known to be strongly associated with corbiculate bees. Bacterial community composition after hibernation compared to before hibernation was characterized by higher OTU richness and evenness, with decreased levels of the core bacteria *Gilliamella* (Proteobacteria, *Orbaceae*) and *Snodgrassella* (Proteobacteria, *Neisseriaceae*), and increased relative abundance of non-core bacteria, including several psychrophilic and psychrotrophic taxa.

## 1. Introduction

Microorganisms are found virtually everywhere and provide numerous benefits to the environment and life on Earth [1,2]. Symbiotic gut bacteria, for example, assist in nutrient acquisition, food digestion, and the protection of their host against pathogens [3,4]. They also influence host behavior, development, reproduction, and overall health [3,5]. In turn, the host provides a nutrient-rich environment that supports the establishment of a microbial community consisting of diverse species acquired through vertical (from mother) or horizontal transmission, and/or from the environment. The exact species composition of these microbial communities is driven by many factors, including host genetics, interactions with the immune system, interactions among members of the microbial community, host diet, and environmental factors such as the pool of external microbes that may invade and stably colonize the insect gut, temperature, nutrient availability, and oxygen level [6].

Many animals, including mammals, reptiles, and amphibians, hibernate when food becomes scarce, allowing them to conserve energy and survive the harsh and food-limited winter conditions [7]. While each animal’s hibernation behavior is different, the effects of hibernation on their metabolism, respiration, and other processes are similar. Typically, hibernation involves fasting, and is characterized by a metabolically depressed state known as torpor, during which body temperature drops to only a few degrees above ambient temperature, and metabolic rates are reduced to 2–4% of normal rates [8,9,10,11,12]. In general, hibernation is associated with increased levels of fat mass and increased abundance of cryoprotectants such as glycerol, sorbitol, and trehalose (or other polyols and sugars) to cope with freezing temperatures [13,14]. From a microbiological point of view, hibernation is known to alter the gut microbiota [10,11,12,14]. For example, in ground squirrels (*Ictidomys tridecemlineatus*), hibernation increases the relative abundance of Bacteroidetes and Verrucomicrobia, which are particular species that are able to survive on host-derived substrates such as mucins. On the other hand, the relative abundance of Firmicutes that presumably rely on the presence of dietary polysaccharides is reduced [11,15]. In larger animals such as brown bears (*Ursus arctos*), hibernation also leads to an increased relative abundance of Bacteroidetes, though not of Verrucomicrobia, and a decrease in Firmicutes and Actinobacteria [12].

To date, little or nothing is known about the effect of hibernation on the gut microbiome of cold-blooded animals such as insects. Many insects have adapted to cold temperatures by entering diapause, which, similar to hibernation in higher animals, is a state of low metabolic activity that is associated with arrested development and increased resistance to environmental stress [16]. A large number of insects overwinter as eggs, which tend to be tolerant to cold and drying. However, some also hibernate as nymphs, larvae, pupae, or adults [17]. Similar to vertebrate species, most hibernating insects empty their gut, reduce body water content, and produce cryoprotectant substances that prevent ice crystals from forming inside their cells [18]. Likewise, significant turnover in the microbial community composition can be expected between individuals before and after hibernation.

In this study, we compared the gut microbiome from midgut to ileum in hibernating queens of buff-tailed bumblebees (*Bombus terrestris*) (*n* = 15) with that of their active counterparts (*n* = 15) using next-generation sequencing of bacterial 16S ribosomal RNA (rRNA) gene amplicons. In nature, bumblebee queens hibernate by digging themselves into the soil, from which they emerge again the following spring to form a new colony [19]. Due to practical reasons (e.g., the difficulty of finding wild bumblebee queens before and immediately after hibernation) as well as to minimize the effect of external factors (e.g., diet, sampling location, environmental pool of microbes), experiments were performed using indoor-reared bumblebees under controlled experimental conditions (artificial hibernation).

## 2. Materials and Methods

### 2.1. Study Species

Experiments were performed using queens of the buff-tailed bumblebee *B. terrestris. Bombus terrestris* is one of the most abundant bumblebee species in Europe, and is known to be an important pollinator of both crops and wild plants [20]. As many other bees, *B. terrestris* produces annual colonies that last for only one year. The colony dies in the fall, while the newly produced queens enter hibernation and start new colonies in the following spring [19]. In order to control experimental conditions, experiments were performed using commercially reared individuals (see below).

### 2.2. Experimental Design

Following mating and a subsequent artificial hibernation period of three months (>80% relative humidity (RH) and 3 °C, no light) at a commercial bumblebee rearing facility (Biobest Group, Westerlo, Belgium), nine *B. terrestris* queens were individually placed in plastic cages of 12 cm × 5.5 cm × 11 cm, allowing them to produce offspring. Each queen was assisted by a callow worker that was manually extracted from the pupal cell, to limit external sources of contamination in the nests. Bees were kept at 28 °C and 55 ± 5% RH in dark conditions, and fed ad libitum with gamma-irradiated pollen and sterile 30% sugar water (2/3 sucrose, 1/6 glucose, 1/6 fructose). Both food sources were refreshed weekly. Once the first daughter queen pupae were present, the full nest (with founder queen, brood, and all colony elements) was transferred to a bigger nest box to allow the sufficient production of new queens in a bigger space. Next, for each nest, daughter queens that had reached the age of three days were collected and put together in a separate nest box, and kept under the same conditions as mentioned above until they were six days old. This step ensured that the new queens were transferred to mating cages at the right age, without compromising the chances of getting contacts with nestmates. A timespan of three days between eclosion and separation of the queens from their nestmates has been shown to be long enough to establish stable gut microbiota through horizontal transmission, via feces secretion and social contacts [21,22,23]. Subsequently, the new queens were mated in sterile cages to increase their survival rate during hibernation and mimic the natural cycle they experience in nature. Next, queens were separated into two groups, including a non-hibernating active group and a hibernating group. While the first was subjected to gut dissection after a short fastening period of one day, during which the bees could empty their gut content (*n* = 15, randomly taken from the produced queens), bees from the hibernating treatment were individually placed in cardboard match boxes and subjected to an artificial hibernation period of 16 weeks at 3 °C without food and light. Immediately thereafter, 15 queens that survived hibernation (*n* = 15, random subsample of all queens that survived hibernation) were dissected using the methodology described below.

### 2.3. Gut Dissection, DNA Extraction, PCR Amplification, and Illumina MiSeq Analysis

Following rinsing with 70% ethanol, each specimen (alive, fresh specimen) was pinned to a polyacrylamide gel plate and immersed in sterile Ringer’s solution. Next, the abdomen was opened by pulling the third segment outward to expose the intestines, and the midgut and ileum were collected into a vial containing one mL of a 40% glycerol solution and homogenized by using zirconia beads and a Fast-Prep24 Instrument (MP Biomedicals, Santa Ana, CA, USA). The dissections were conducted with the help of a binocular microscope (Wild M420 Makroskop, Wild, Heerbrugg, Switzerland). After every dissection, Ringer’s solution was replaced, and the gel plate was sterilized with 70% ethanol. Samples were preserved at −80 °C.

In order to extract DNA from the gut homogenates, the gut was crushed in a 170-µL lysozyme solution (100 mg/mL), and DNA was extracted according to Meeus et al. [21]. A negative control was included during extraction in which the lysozyme solution without gut material was used as the starting material. DNA samples were then subjected to PCR amplification using sample-specific barcode-labeled versions of the primers 515F (5’-GTGCCAGCMGCCGCGGTAA-3’) and 806R (5’-GGACTACHVGGGTWTCTAAT-3’), generating amplicons covering the hypervariable V4 region of the bacterial 16S rRNA gene [24,25] (Table S1, Supporting Information). Again, a negative control was included (PCR amplification control), this time by replacing template DNA with sterile water. Results obtained for both types of negative controls were satisfactory, confirming that the experimental conditions were met to achieve robust data. Amplification was performed in a reaction volume of 40 µL containing 1× Titanium Taq PCR buffer, 150 μM of each dNTP, 0.5 μM of each primer, 1× Titanium Taq DNA polymerase (Clontech, Saint-Germain-en-Laye, France), and two µL DNA (5 ng µL^−1^). The reaction was initiated by denaturation at 94 °C for 120 s, followed by 30 cycles of denaturation at 94 °C for 45 s, annealing at 59 °C for 45 s, and elongation at 72 °C for 45 s, followed by a final elongation at 72 °C for 10 min. Amplicons were then purified using Agencourt AMPure XP magnetic beads (Beckman Coulter Genomics GmbH, South Plainfield, UK) according to the manufacturer’s instructions. Following quantification of the purified products using a Qubit High Sensitivity Fluorometer kit (Invitrogen, Carlsbad, CA, USA) amplicons were combined at equimolar concentrations into an amplicon library. Subsequently, the library was subjected to an ethanol precipitation and loaded on an agarose gel. Next, the band of the expected size (*c*. 250 bp) was excised, and the DNA was purified again, this time using the QIAquick Gel Extraction Kit (Qiagen, Hilden, Germany). Finally, the DNA concentration was measured again, and the library was diluted to two nM and sequenced (together with a number of other samples) at the Center for Medical Genetics (University of Antwerp, Antwerp, Belgium) using an Illumina MiSeq sequencer with a v2 500-cycle reagent kit (Illumina, San Diego, CA, USA).

Sequences were received as a demultiplexed FASTQ file. Paired-end reads were merged using USEARCH (v10.0.240) to form consensus sequences [26] and truncated at the 250th base. Shorter reads or reads with a total expected error threshold above 0.05 were discarded using VSEARCH v2.4.0 [27]. The “classify.seqs” and “remove.lineage” commands in Mothur (v1.36.1) and the Silva database (v1.23) were used to identify and remove potential mitochondrial, chloroplast, archaeal, and eukaryote DNA sequences that may have been co-amplified by the primers. Next, sequences were grouped into operational taxonomic units (OTUs) based on a 3% sequence dissimilarity cut-off using the UPARSE greedy algorithm in USEARCH, during which chimeric sequences were also removed [26]. Further, OTUs were filtered to retain only OTUs with a relative abundance of ≥0.05% in at least one sample [28]. Subsequently, the taxonomic origin of each remaining OTU was determined with the SINTAX algorithm implemented in USEARCH [29], based on the Silva Living Tree Project v1.23 database [30]. In general, taxonomic assignments can be considered reliable when bootstrap confidence values exceed 0.80. Furthermore, BLAST (basic local alignment search tool) searches were performed against type materials in GenBank, verifying the identity of the most important OTUs. Additionally, for core bacteria (i.e., bacteria that have been repeatedly associated with individuals of *Bombus* bumblebees), identifications were refined by available information in the literature and GenBank [31,32]. Raw sequence data were deposited in the NCBI SRA database under BioProject accession PRJNA438866.

### 2.4. Determination of Bacterial Load and Pathogen Infection Using qPCR

Quantitative real-time PCR (qPCR) was used to estimate total bacterial abundance in the gut samples, as well as assess the presence and abundance of two widespread bumblebee pathogens, i.e., the microsporidian parasite *Nosema bombi* (Microsporidia, *Nosematidae*) and the trypanosome *Crithidia bombi* (Kinetoplastida, *Trypanosomatidae*). Previous research has shown that infection by these pathogens may be related to the gut community composition [19,31,33]. Therefore, when investigating microbial gut communities in bees, it is important to know whether or not pathogen infection occurred. Specifically, the universal bacterial primers 519F/907R [34] were used to amplify total copies of the 16S rRNA gene. Additionally, 211F/211R and 119F/119R were used for the detection and quantification of *N. bombi* and *C. bombi*, respectively [35]. qPCR amplifications were performed in MicroAmp Fast 8-Tube Strips (Life Technologies, Carlsbad, CA, USA) using a StepOnePlus real-time PCR system (Applied Biosystems, Carlsbad, CA, USA), and each reaction was performed in duplicate. Each reaction contained 1.0 µL (5 ng) DNA, 10.0 µL of the iTaq Universal SYBRGreen supermix (Bio-Rad, Hercules, CA, USA), 0.2 µL of each primer (20 µM), and 8.6 µL of sterile water. Thermal cycling conditions consisted of two minutes at 95 °C, followed by 40 amplification cycles of 15 s at 95 °C, 30 s at 59 °C (519F/907R) or 64.5 °C (211F/211R and 119F/119R), and 30 s at 60 °C. In each analysis, a positive and negative control (template DNA replaced by sterile water) was included. Quantification was based on standard curves from the amplification of cloned target sequences in a TOPO-TA vector (Invitrogen).

### 2.5. Statistical Analyses

For each sample, a rarefaction curve was constructed using the Vegan package (v2.4-6) for R [36]. Additionally, OTU richness (*S*) was determined for each specimen by counting the number of observed OTUs, and bacterial diversity was approximated by the Shannon diversity index (*H*) and Pielou’s evenness (*J* = *H*/ln(*S*)). Shannon diversity was exponentially transformed (exp(*H*)), by which the variable behaves in a linear manner, in contrast to the non-transformed Shannon diversity [37]. The richness, exp(*H*), and evenness of each group of bees was determined as the average of the richness, exp(*H*), and evenness of the 15 bee samples, respectively, and compared between treatments by a simple *t*-test. Non-metric multidimensional scaling (NMDS) was used to visualize the level of similarity in community composition between the different samples based on Bray–Curtis similarities (i.e., based on relative abundance data). Further, using the same distance matrix, UPGMA (unweighted pair group method with arithmetic mean) clustering was used to generate a dendrogram. Permutational multivariate analysis of variance (PERMANOVA) using the anosim function in the vegan package was performed to test for significant differences in gut microbial community composition between individuals before and after hibernation. Furthermore, β-diversity was calculated using the weighted UniFrac distance metric, and significant differences across both bee groups were again evaluated using PERMANOVA. Finally, an indicator species analysis (ISA) was performed using the Indicspecies package (v1.7-1) in R [36] to identify microbial OTUs that were significantly associated with one of both groups of bumblebee queens. Indicator species values are based on how specific and widespread an OTU is within a particular group, and are independent of the relative abundance of other bacteria. Relative abundance of the observed OTUs was represented in bar charts. Additionally, data were parsed using the Circos table viewer [38].

## 3. Results

High-throughput 16S rRNA gene sequencing and subsequent bioinformatics analysis yielded a dataset of 698,172 sequences (ranging between 22,266–23,357 sequences per sample) that could be classified into 674 bacterial OTUs (Table S2, Supporting Information). In general, rarefaction curves approached saturation, or tended to approach saturation (Figure S1, Supporting Information). Furthermore, there was no correlation between sequence depths and diversity variables, indicating that the bacterial communities could be accurately compared at the obtained sequence depths. qPCR analysis showed similar amounts of 16S rRNA gene copies in the queens before and after hibernation (on average 4 × 10^8^ and 2 × 10^8^ gene copies before and after hibernation, respectively). None of the specimens investigated was found to be infected by the bumblebee pathogens *N. bombi* or *C. bombi*.

**Figure 1:**
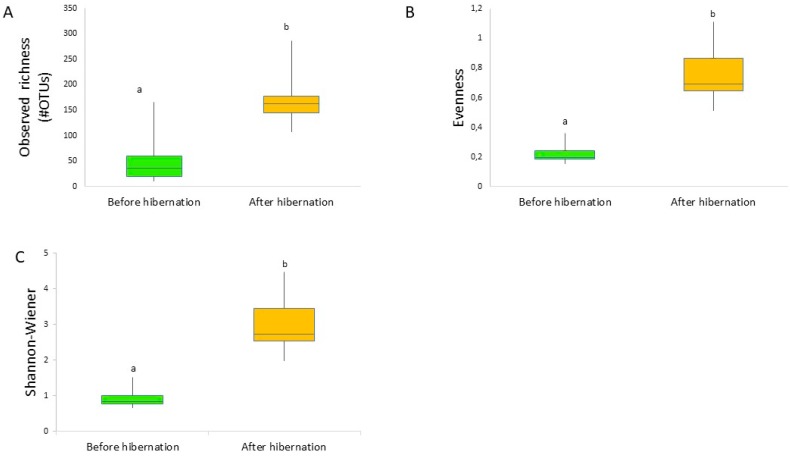
Observed richness (**A**), evenness (**B**), and Shannon–Wiener diversity (**C**) boxplots of the gut bacteria occurring in the midgut and/or ileum in indoor-reared bumblebee queens (*Bombus terrestris*) before (*n* = 15) and after hibernation (*n* = 15). The boxplots show the upper and lower quartiles; the whiskers indicate variability outside the upper and lower quartiles. Further, the median is plotted. Diversity indices were significantly different (*p* < 0.05) between treatments (Shannon–Wiener values were first exponentially transformed before performing the statistical analysis).

**Figure 2:**
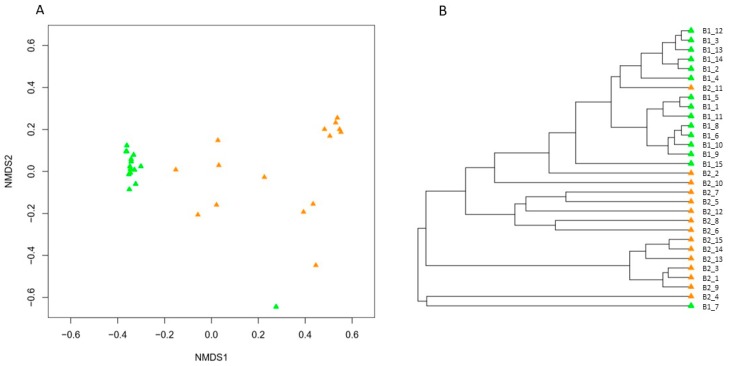
(**A**) Non-metric multidimensional scaling (NMDS) ordination (stress value = 0.09) based on Bray–Curtis similarities depicting the gut (midgut and ileum) bacterial community composition of indoor-reared bumblebee queens (*Bombus terrestris*) before (green; *n* = 15) and after hibernation (orange; *n* = 15). The distance between different points on the plot reflects the similarity level in bacterial community composition: the more similar the bacterial communities, the smaller the distance between the points. (**B**) UPGMA (Unweighted pair group method with arithmetic mean) dendrogram visualization of the clustering analysis.

**Figure 3:**
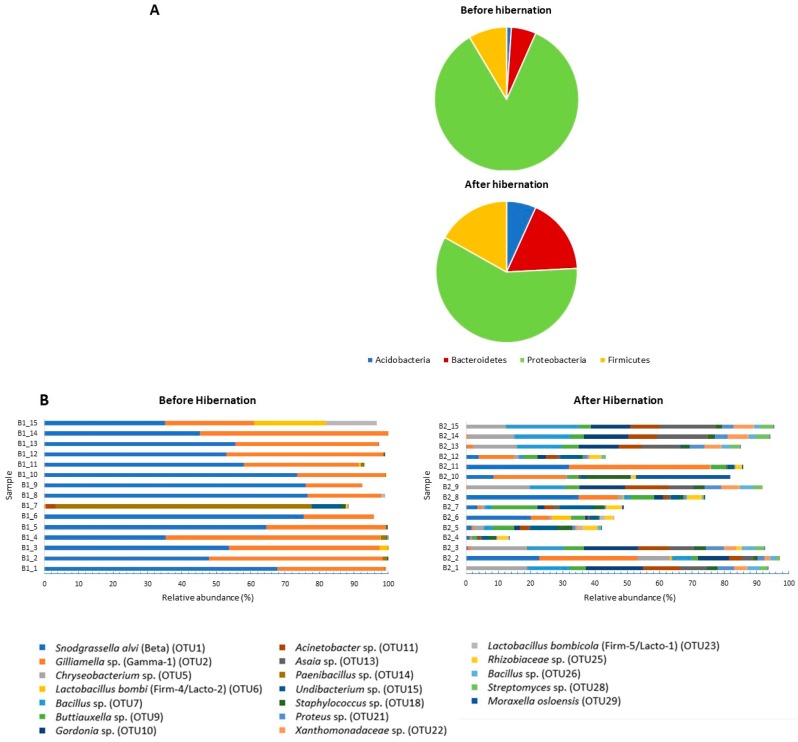
(**A**) Relative abundance (%) of the bacterial phyla found in the midgut and/or ileum of indoor-reared bumblebee queens (*Bombus terrestris*) before (*n* = 15) and after hibernation (*n* = 15). (**B**) Gut bacterial community composition at the level of operational taxonomic units (OTUs). Only the most abundant OTUs (i.e., with a mean sequence relative abundance (*n* = 30) >1%) are represented in the figure. OTUs were identified by a BLAST (basic local alignment search tool) search against type materials in GenBank and identified up to species level if only one top hit was obtained. Note that the *Paenibacillus* OTU (OTU14) is not related to *Paenibacillus larvae*, which is a species lethal to honey bee and bumblebee larvae. The highest sequence similarity was found with type strains of *Paenibacillus amylolyticus*, *P. pabuli*, *P. taichungensis*, *P. tundra*, *P. tylopili*, *P. xylanexedens*, and *P. xylanilyticus* (for all 100% sequence identity on a total of 250 bp).

Diversity indices were significantly (*p* < 0.05) higher for the bees after hibernation compared to before hibernation (Figure 1; Table S3, Supporting Information). Observed OTU richness varied between 11–130 OTUs per bumblebee before hibernation (average: 42 OTUs), while after hibernation, queens contained between 82–323 OTUs (average: 154 OTUs) (Figure 1; Table S3, Supporting Information). NMDS ordination (Bray–Curtis; stress = 0.09) and UPGMA clustering showed a clear separation of both groups of samples, with minimal overlap (Figure 2). Anosim analysis further revealed a significant difference in community composition (*R* = 0.6366, *p* = 0.0001; the closer R is toward 1, the more dissimilar the two groups of samples). More specifically, hibernation significantly increased the relative abundance of Acidobacteria (from 1.1% to 6.3%), Bacteroidetes (from 5.5% to 16.2%), and Firmicutes (from 8.5% to 15.7%), while a relative abundance of Proteobacteria was reduced (from 84.2% to 54.8%), especially due to lowered levels of *Snodgrassella* and *Gilliamella* (Figure 3). Results were confirmed by weighted UniFrac analysis taking phylogenetic relationships among members of the microbial community into account, weighted by OTU abundance (Anosim: *R* = 0.7083, *p* = 0.0001; Figure S2, Supporting Information).

ISA revealed the presence of nine indicator OTUs which were significantly (Indicator value >0.25 and *p* < 0.05) attributed to hibernating bumblebees. These were OTU5 (*Chryseobacterium* sp.; *Flavobacteriaceae*; Bacteroidetes), OTU7 (*Bacillus* sp.; *Bacillaceae*; Firmicutes), OTU9 (*Buttiauxella* sp.; Enterobacteriaceae; Proteobacteria), OTU10 (*Gordonia* sp.; *Nocardiaceae*; Proteobacteria), OTU11 (*Acinetobacter* sp.; *Moraxellaceae*; Proteobacteria), OTU13 (*Asaia* sp.; *Acetobacteraceae*; Proteobacteria), OTU18 (*Staphylococcus* sp.; *Staphylococcaceae*; Firmicutes), OTU 21 (*Proteus* sp.; Enterobacteriaceae; Proteobacteria), and OTU 22 (*Xanthomonadaceae* species; Proteobacteria). No OTUs were significantly associated with the before hibernation treatment (Table 1; see Figure S3 (Supporting Information) for box plots representing relative abundances). In total, two OTUs were shared between all of the tested individuals, irrespective of treatment, i.e., the core bacteria *Snodgrassella* (OTU1; *Snodgrassella alvi*; *Neisseriaceae*; Betaproteobacteria; also known as phylotype “Beta” [32]) and *Gilliamella* (OTU2; *Orbaceae*; Gammaproteobacteria; also known as phylotype “Gamma-1” [32]). Likewise, the bee-specific lactobacilli *Lactobacillus bombi* (OTU6; also known as phylotype “Firm-4/Lacto-2” [32]) and *Lactobacillus bombicola* (OTU23; phylotype “Firm-5/Lacto-1” [32]) were commonly found in the specimens investigated (Table 1).

**Figure 4:**
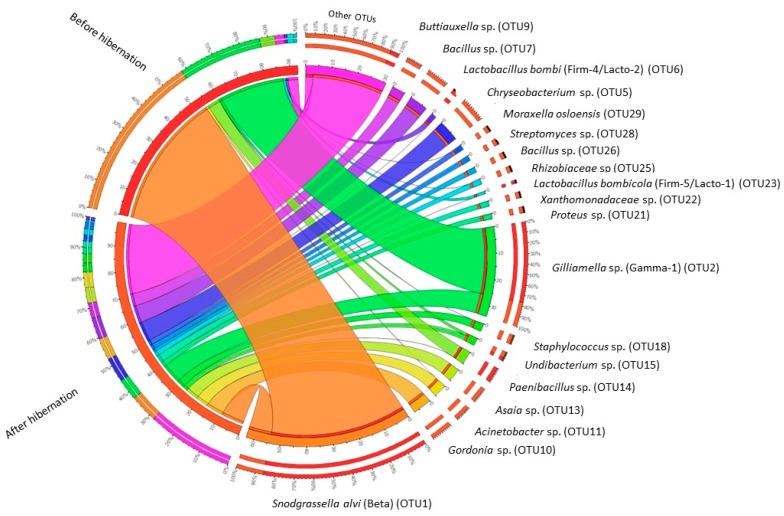
Circular visualization of the occurrence and abundance of gut bacterial operational taxonomic units (OTUs) in bumblebee queens (*Bombus terrestris*) before (*n* = 15) and after hibernation (*n* = 15). Thickness of the ribbon refers to the number of reads assigned to a treatment. The outer ring summarizes the relative abundance of OTUs. Only the most abundant OTUs (i.e., with a mean relative abundance (*n* = 30) ≥1%) are represented in the figure. All other OTUs were grouped together in “Other OTUs”. OTUs were identified by a BLAST search against type materials in GenBank, and identified up to species level if only one top hit was obtained. Data was parsed with Circos table viewer [38]. Note that the *Paenibacillus* OTU (OTU14) is not related to *Paenibacillus larvae*, which is a species that is lethal to honey bee and bumblebee larvae. The highest sequence similarity was found with type strains of *Paenibacillus amylolyticus*, *P. pabuli*, *P. taichungensis*, *P. tundra*, *P. tylopili*, *P. xylanexedens*, and *P. xylanilyticus* (for all 100% sequence identity on a total of 250 bp).

When zooming in on the most abundant community members, a total of 19 OTUs were found with a mean relative abundance ≥1% (*n* = 30). All of them were found in the hibernating queens (accounting for 71.5% of the sequences); a subset of 13 OTUs was found before hibernation (90.0% of sequences) (Table 1). As can be observed from Figure 3 and Figure 4, the gut bacterial communities of active bumblebee queens were mainly dominated by *Snodgrassella* (OTU1; mean read abundance of 54.5%) and *Gilliamella* (OTU2; 33.8%) (Figure 3 and Figure 4). In contrast, the OTUs in hibernating queens were more evenly distributed and occurred at a relative abundance of less than 9% (e.g., 8.7% for the *Snodgrassella* OTU and 8.8% for *Gilliamella*). Besides *Snodgrassella* and *Gilliamella*, queens after hibernation mainly contained OTU5 (*Chryseobacterium* sp.; 7.6%), OTU10 (*Gordonia* sp.; 7.1%), OTU7 (*Bacillus* sp.; 7.0%), OTU9 (*Buttiauxella* sp. (Gamma-E1); 5.1%), OTU13 (*Asaia* sp.; 5.1%), and OTU11 (*Acinetobacter* sp.; 5.0%), many of which were an indicator of OTU for hibernation and were not or are only sporadically detected in active queens (Table 1).

## 4. Discussion

### 4.1. Community Structure of the Gut Bacterial Microbiota in Indoor-Reared Bumblebee Queens

Deep sequencing of the microbiota occurring in the midgut and/or ileum of indoor-reared queens of *B. terrestris*, which is one of the most common bumblebees in Europe, revealed several bacterial taxa that are known to be associated with corbiculate bees. *Snodgrassella*, *Gilliamella,* and *Lactobacillus* have been described as the core gut bacteria of *Apis* [39] and workers of both wild and indoor-reared *B. terrestris* [32]. Additionally, bifidobacteria and Bacteroidetes are associated with honeybees and bumblebees, but with a more irregular occurrence [19,32,40]. Our results are in line with these previous findings, as *Snodgrassella* (OTU1; Beta) and *Gilliamella* (OTU2; Gamma-1) both occurred in the gut of every specimen investigated. Additionally, lactobacilli were found in almost every sample (27 out of 30 specimens). Specifically, the bee-specific lactobacilli *L. bombi* (OTU6; Firm-4/Lacto-2) and *L. bombicola* (OTU23; Firm-5/Lacto-1) were frequently found, i.e., in 19 and 18 of the 30 queens investigated, respectively. In addition, OTU194 corresponding to *Lactobacillus apis* (previously found in honeybees [41]) was found in 15 samples. Further, a number of environmental lactobacilli were detected, albeit more sporadically (Table S2, Supporting Information).

In contrast to Meeus et al. [32], who found *Bifidobacteriaceae* (albeit at low relative abundance) in the majority of *B. terrestris* workers investigated (22 out of 24 samples), bifidobacteria were only found in half of the specimens investigated here, with the bee-associated phylotype Bifido-3 (OTU24; *Bombiscardovia coagulans;* [32]) as the most prevalent OTU (present in 12 out of 30 investigated specimens). Additionally, other *Bifidobacteriaceae* OTUs occurred in a number of samples, including an OTU corresponding to *Bifidobacterium animalis* (OTU231; found in five samples), and another OTU corresponding to *Bifidobacterium commune* (OTU75; found in three samples). While *B. animalis* is commonly found in the animal intestinal environment, *B. commune* has only recently been described as a novel species inhabiting the bumblebee gut [42]. Most probably, the low prevalence of bifidobacteria can be explained by our focus on the midgut and ileum, while other studies investigated the microbiome of whole guts, including rectum [19,32,39]. Previous research has shown that the midgut of social bees only contains a few bacteria, while the ileum and rectum are strongly colonized by bacteria, totaling up to 10^8^ and 10^9^ bacterial cells, respectively [43,44]. Furthermore, while the ileum is dominated by *Snodgrassella*, *Gilliamella*, and the lactobacilli Firm-4/Lacto-2 and Firm-5/Lacto-1, the rectum is dominated by lactobacilli and bifidobacteria [43,44]. Therefore, as the rectum was excluded in our analysis, this may explain the low prevalence and abundance of Bifidobacteriaceae.

Altogether, these results indicate that the gut microbiome of bumblebees is similar to that of honeybees and stingless bees, which are also social and contain gut communities dominated by some of the same core bacterial species as those found in bumblebees [44]. Phylogenetic analyses of strains from diverse corbiculate bee species suggest that these core species colonized a common ancestor of the corbiculate clade about 80 million years ago, and that the strains subsequently diversified, with some host lineages acquiring a number of additional bacterial phylotypes [45]. Although these bees go through a different life cycle, all of them are social and live in colonies consisting of a queen and workers, enabling the transmission of gut microbiota through social contacts.

### 4.2. Impact of Hibernation on the Gut Bacterial Community Composition

Our results further indicate that the hibernation of bumblebee queens leads to significantly altered gut communities that are characterized by a different bacterial community structure, higher overall diversity, and higher evenness. Hibernation significantly increased the relative abundance of Bacteroidetes (+10.7%) and Firmicutes (+7.2%), while the relative abundance of Proteobacteria drastically decreased (−29.4%). This is partially consistent with previous findings in hibernating mammals, where an increase in the relative abundance of Bacteroidetes was found, but in contrast to our results, there was also a reduction of Firmicutes [11,12]. In contrast to our results, studies on hibernating mammals and amphibians found a reduction in gut bacterial diversity after hibernation [12,46,47].

The increased diversity that was observed in our study can most probably be explained by the core gut residents *Snodgrassella* and *Gilliamella*, which were abundantly present before hibernation, perform worse during hibernation (decrease in relative abundance from over 54% and 33% to less than 9%, respectively), thereby facilitating the growth of several other bacteria that are better adapted to thrive under the harsh conditions of hibernation (e.g., poor nutrients, low temperature). Indeed, hibernating bumblebees contained a wide diversity of bacteria, including several facultative psychrophilic (cold-loving) and psychrotrophic (cold-tolerant) bacteria [48] that were almost not found before hibernation. These included, for example, members of the genera *Acinetobacter*, *Buttiauxella*, *Chryseobacterium*, *Hafnia*, *Psychrobacter,* and *Pseudomonas* (Table S2, Supporting Information). Bacteria such as *Gilliamella* and *Snodgrassella* are mesophilic bacteria that grow best at moderate temperatures and perform worse at cold termperatures [49]. However, because we investigated the gut microbiome from the midgut until the ileum, it remains to be investigated whether the same trends will be observed when the rectum is taken into account.

Our data also suggest that the bacteria found after hibernation were also present in the pre-hibernation queens, but occurred at a low relative abundance, and were therefore not detected or not frequently detected by the amplicon sequencing approach (Table S2, Supporting Information). Sensitive qPCR assays assessing absolute numbers of these taxa may be helpful to confirm this. Further research is also needed to unravel the exact mechanisms that affect the microbial community structure through hibernation, and from where these environmental microbes come from when bumblebees were exposed to sterile food and incubated in a sterile environment. Potentially, insect boxes or food sources became contaminated over the duration of the experiment.

Due to practical limitations, we were not able to control for the factor of time (age), which also may have had an effect on gut microbiota as seen in honeybee queens [49]. *Bombus terrestris* queens generally die after a period of six weeks if they do not hibernate (Pozo et al., unpublished results). Therefore, non-hibernating queens of the same age as those investigated after a 16-week hibernation period could not be included as a control group. It also has to be noted that our experiments were performed using commercially reared bumblebees that were forced to undergo hibernation under controlled experimental conditions. We specifically used indoor-reared specimens to exclude genetic and external factors as much as possible. It remains to be investigated whether the same effects are observed in wild populations that face less sterile conditions in a fluctuating, more complex environment. Previous research has shown higher gut microbiome diversity in wild *B. terrestris* workers with more non-core bacteria compared to indoor-reared specimens [32]. Therefore, it may be hypothesized that environmental conditions have a significant impact on the gut communities of hibernating wild colonies. Further research is needed to fully investigate this possibility.

## 5. Conclusions

In conclusion, our study showed that the bacterial community composition of bumblebee queens after hibernation compared to before hibernation was characterized by higher OTU richness and evenness. Hibernation led to decreased levels of the core bacteria *Gilliamella* and *Snodgrassella*, and increased relative abundance of non-core bacteria, including several psychrophilic and psychrotrophic taxa. Most probably this can be explained because these core bacteria perform worse during hibernation, thereby facilitating the growth of other bacteria that are better adapted to thrive under the harsh conditions of hibernation.

## Figures and Tables

**Table 1:** Mean relative abundance and prevalence in indoor-reared *Bombus terrestris* queens (*n* = 15 per treatment) of the main operational taxonomic units (OTUs) found in this study ^a^.

OUT ^b^	Taxonomic Affiliation				Before Hibernation		After Hibernation	
	Phylum	Family	Species ^c^	Name OTU in Literature ^d^	Relative Abundance (%)	Present in *B. terrestris* (*n* = 15)	Relative Abundance (%)	Present in *B. terrestris* (*n* = 15)
OTU_1	Proteobacteria	*Neisseriaceae*	*Snodgrassella alvi* (98.8%)	Beta	54.525	15	8.668	15
OTU_2	Proteobacteria	*Orbaceae*	*Gilliamella apicola, G. bombi, G. bombicola; G. mensalis* (100%)	Gamma-1	33.805	15	8.753	15
OTU_5 *	Bacteroidetes	*Flavobacteriaceae*	*Chryseobacterium daecheongense* (100%)		0.001	2	7.593	15
OTU_10 *	Actinobacteria	*Nocardiaceae*	*Gordonia polyisoprenivorans, G. soli* (100%)		0	0	7.131	12
OTU_7 *	Firmicutes	*Bacillaceae*	Several *Bacillaceae* spp. (100%)		0.001	2	6.999	15
OTU_14	Firmicutes	*Paenibacillaceae*	*Paenibacillus amylolyticus, P. pabuli, P. taichungensis, P. tundra, P. tylopili, P. xylanexedens and P. xylanilyticus* (100%)		5.3	15	0.116	13
OTU_9 *	Proteobacteria	*Enterobacteriaceae*	Several *Enterobacteriaceae* spp., including *Buttiauxella agrestis* (100%)	Gamma-E1	0.004	7	5.124	15
OTU_13 *	Proteobacteria	*Acetobacteraceae*	*Asia bogorensis, A. siamensis, A. prunella, A. lannensis* (100%)		0	0	5.112	15
OTU_11 *	Proteobacteria	*Moraxellaceae*	*Acinetobacter vivianii, A. proteolyticus, A. modestus, A. courvalinii* (100%)		0.186	7	4.996	14
OTU_18 *	Firmicutes	*Staphylococcaceae*	Several *Staphylococcaceae* spp. (100%)		0.111	9	2.954	15
OTU_15	Proteobacteria	*Oxalobacteraceae*	*Undibacterium oligocarboniphilum* (100%)		0.618	12	2.417	15
OTU_21 *	Proteobacteria	*Enterobacteriaceae*	*Proteus mirabilis, P. penneri, Cosenzaea myxofaciens* (100%)		0	0	2.340	15
OTU_22 *	Proteobacteria	*Xanthomonadaceae*	*Thermomonas haemolytica* (99%)		0	0	2.287	14
OTU_29	Proteobacteria	*Moraxellaceae*	*Moraxella osloensis* (100%)		0.007	4	2.064	11
OTU_25	Proteobacteria	*Rhizobiaceae*	*Agrobacterium tumefaciens, A. fabrum* (100%)		0.049	7	1.958	15
OTU_6	Firmicutes	*Lactobacillaceae*	*Lactobacillus bombi* (100%)	Firm-4/Lacto-2	1.612	9	0.507	10
OTU_28	Actinobacteria	*Streptomycetaceae*	Several *Streptomycetaceae* spp. (100%)		0	0	1.567	14
OTU_26	Firmicutes	*Bacillaceae*	Several *Bacillaceae* spp. (100%)		0	0	1.518	13
OTU_23	Firmicutes	*Lactobacillaceae*	*Lactobacillus bombicola* (100%)	Firm-5/Lacto-1	1.092	11	0.213	7

^a^ Only OTUs with a mean sequence relative abundance (*n* = 15) ≥1% are represented in the Table; ^b^ Indicator OTUs significantly associated with hibernating queens are indicated with an asterisk (determined by indicator species analysis; indicator value >0.25 and *p* < 0.05). No OTUs were significantly associated with the treatment before hibernation; ^c^ Nearest neighbor based on a BLAST (Basic local alignment search tool) search in GenBank against type strains. Percentage of sequence identity (on a total of 250 bp) is reported between brackets; ^d^ As used by Meeus et al. (2015).

## Supplementary Materials

The following are available online at http://www.mdpi.com/2075-4450/9/4/188/s1, Figure S1: Rarefaction curves showing the number of gut bacterial operational taxonomic units (OTUs) per bumblebee queen (*Bombus terrestris*) before (green; *n* = 15) and after hibernation (orange; *n* = 15). Rarefaction curves reached saturation, suggesting that the most abundant community members were covered by our sequencing depth; Figure S2: (A) Non-metric multidimensional scaling (NMDS) plot (stress value = 0.08) based on the weighted UniFrac distance matrix depicting the gut (midgut and ileum) bacterial community composition of indoor-reared bumblebee queens (*Bombus terrestris*) before (green; *n* = 15) and after hibernation (orange; *n* = 15)). The distance between different points on the plot reflects the similarity level in bacterial community composition: the more similar the bacterial communities, the smaller the distance between the points. (B) UPGMA (unweighted pair group method with arithmetic mean) dendrogram visualization of the clustering analysis; Figure S3: Box plots showing the read abundance (number of reads) of gut bacterial operational taxonomic units (OTUs) significantly associated (Indicator value > 0.25 and *p* < 0.05) with hibernating bumble bee queens (*Bombus terrestris*) before (*n* = 15) and after hibernation (*n* = 15). The boxplots show the upper and lower quartiles; the whiskers indicate variability outside the upper and lower quartiles. Further, the median is plotted; Table S1: Primer design and sample-specific barcodes; Table S2: Identification of operational taxonomic units (OTUs) according to the Silva v1.23 database and distribution over the investigated samples; Table S3: Bacterial community diversity indices and 16S rRNA gene copy numbers for the bumblebee queens (*Bombus terrestris*) investigated in this study.
