# Supplementary material for: Hibernation Leads to Altered Gut Communities in Bumblebee Queens (Bombus terrestris)
Source: Insects. 2018 Dec 7;9(4):188. doi: 10.3390/insects9040188 (PMC6316087; doi:10.3390/insects9040188)
Supplement: Supplementary file 1 [file insects-09-00188-s001.zip › Supplementary/Supplemetary-f.docx]

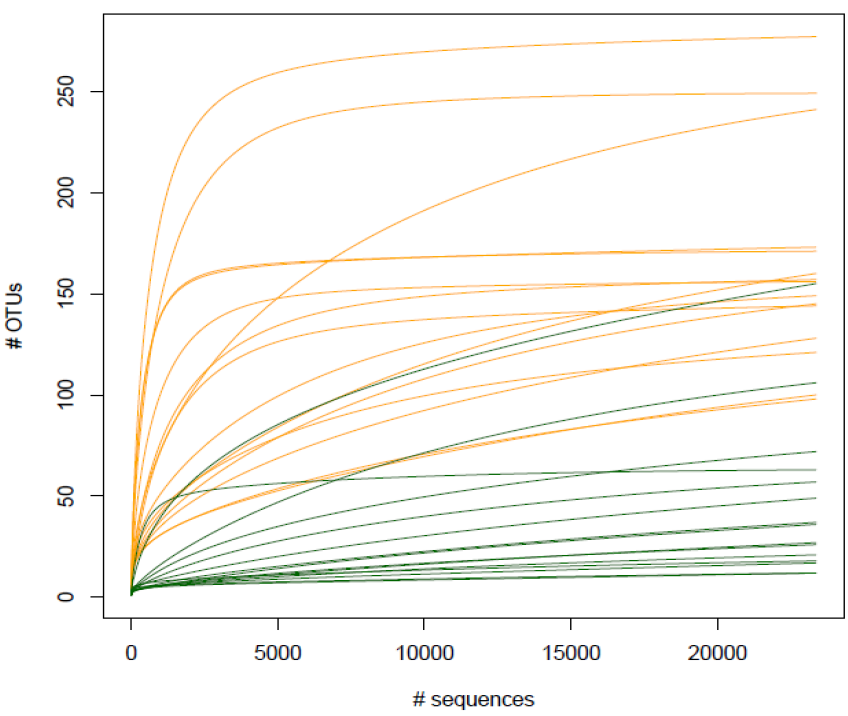


Figure S1: Rarefaction curves showing the number of gut bacterial operational taxonomic units (OTUs) per bumblebee queen (*Bombus terrestris*) before (green; *n* = 15) and after hibernation (orange; *n* =15). Rarefaction curves reached saturation, suggesting that the most abundant community members were covered by our sequencing depth.


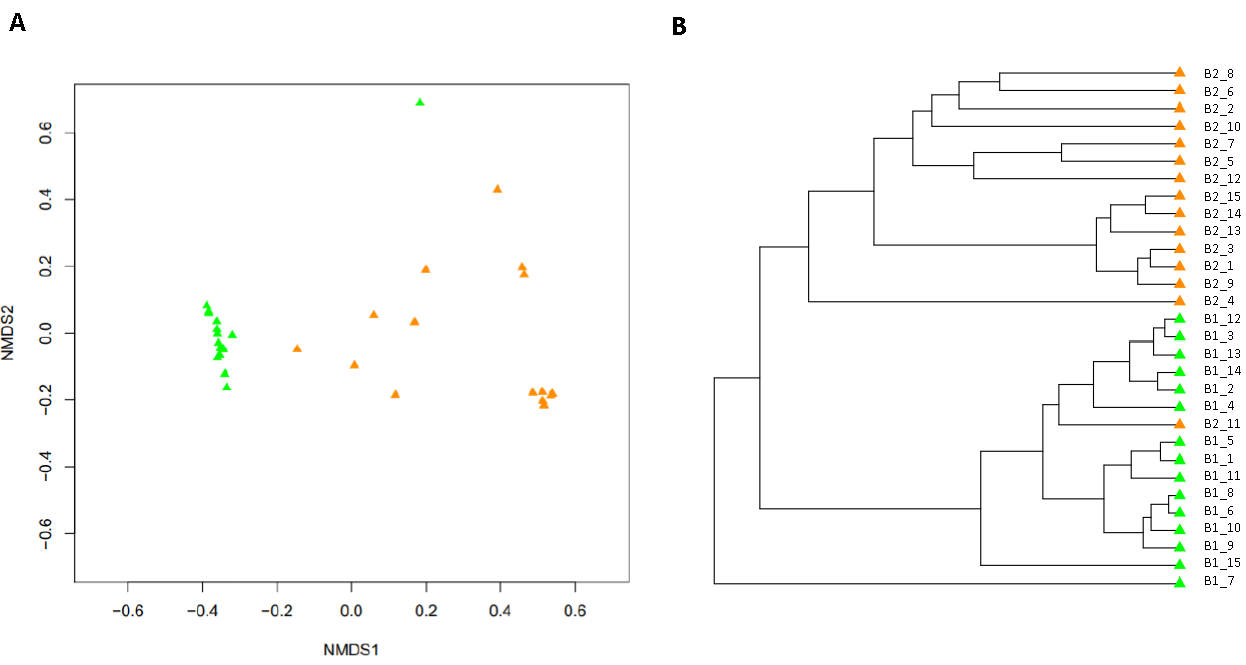


Figure S2: (A) Non-metric multidimensional scaling (NMDS) plot (stress value = 0.08) based on the weighted UniFrac distance matrix depicting the gut (midgut and ileum) bacterial community composition of indoor-reared bumblebee queens (*Bombus terrestris*) before (green; *n* = 15) and after hibernation (orange; *n* = 15)). The distance between different points on the plot reflects the similarity level in bacterial community composition: the more similar the bacterial communities, the smaller the distance between the points. (B) UPGMA (unweighted pair group method with arithmetic mean) dendrogram visualization of the clustering analysis.


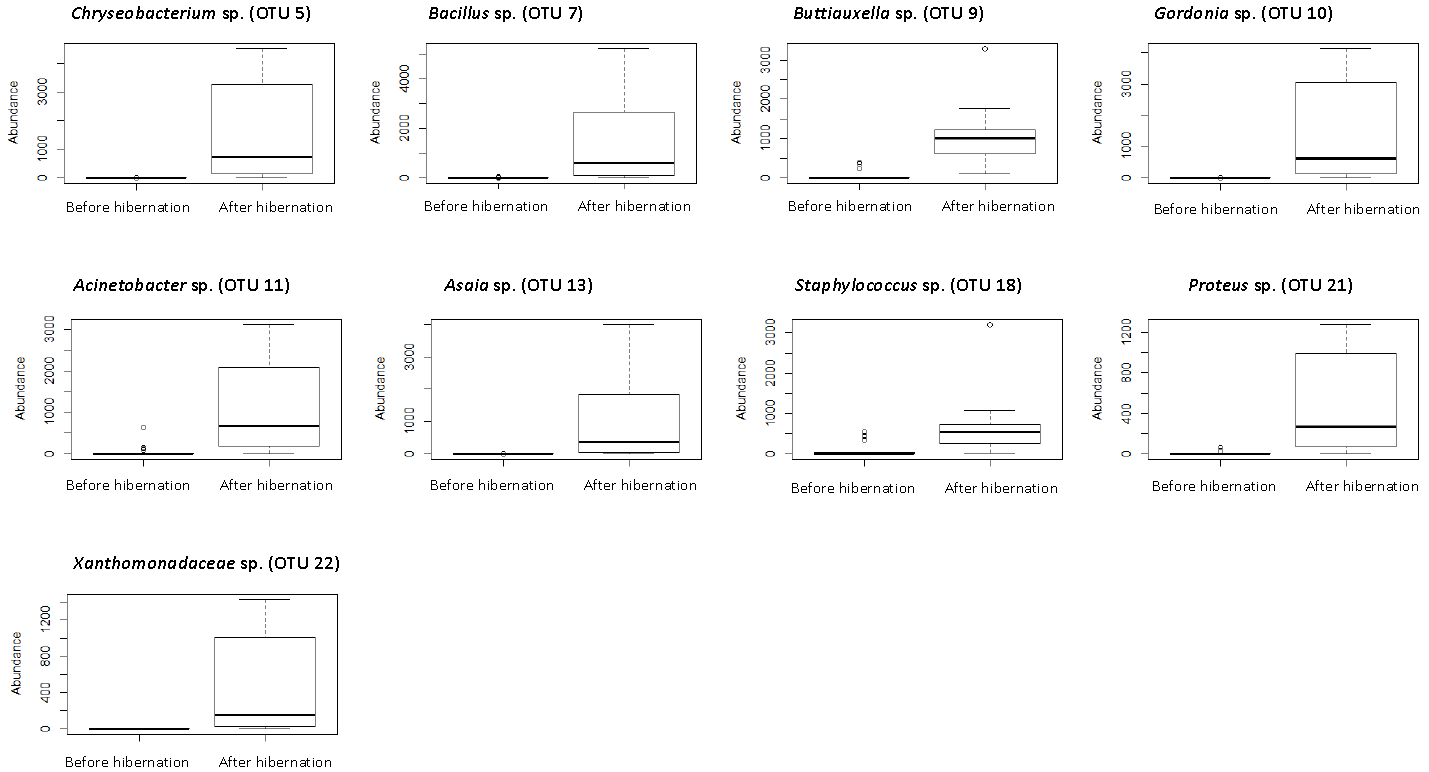


Figure S3: Box plots showing the read abundance (number of reads) of gut bacterial operational taxonomic units (OTUs) significantly associated (Indicator value > 0.25 and *P* < 0.05) with hibernating bumble bee queens (*Bombus terrestris*) before (*n* = 15) and after hibernation (*n* = 15). The boxplots show the upper and lower quartiles; the whiskers indicate variability outside the upper and lower quartiles. Further, the median is plotted;
